# Cleft Lip and Palate Research Trends in Saudi Arabia: A Bibliometric Analysis

**DOI:** 10.7759/cureus.52085

**Published:** 2024-01-11

**Authors:** Hatem H Hamadallah, Khalid N Alturki, Mahmoud Alsulaimani, Ahmad Othman, Abdulaziz O Altamimi

**Affiliations:** 1 Dentistry, Taibah University, Madinah, SAU; 2 Orthodontics and Dentofacial Orthopedics, Taibah University, Madinah, SAU; 3 Oral and Maxillofacial Surgery, Taibah University, Madinah, SAU; 4 Endodontics, Dental Center, Ohud Hospital, Madinah, SAU

**Keywords:** publication patterns, saudi arabia, research trends, bibliometric analysis, cleft lip and palate

## Abstract

As Saudi Arabia advances in medical education and patient care, assessing its contribution to cleft lip and palate (CLP) research is vital. This bibliometric analysis aims to map the trends, collaborations, and impact of Saudi research in CLP. Utilizing the Web of Science database, this study conducted a comprehensive bibliometric analysis of CLP research related to Saudi Arabian publications. The analysis included data extraction and assessment of publications, citations, H-index, and international collaborations using advanced bibliometric tools and software. A total of 89 CLP-related articles in Saudi Arabia were retrieved. The findings indicated a steady increase in publications and citations over the years, reflecting growing interest and recognition of CLP's challenges in the Saudi healthcare context. King Abdulaziz University and King Saud University emerged as leading contributors. International collaboration was evident, with significant partnerships with countries like the USA, Canada, the UK, and others. The Cleft Palate-Craniofacial Journal and the Saudi Dental Journal were identified as the most influential journals in disseminating Saudi CLP research. The study highlights a positive growth trajectory in Saudi CLP research, marked by increased publications, citations, and international collaborations. It underscores the importance of continuous research and the need for enhanced efforts to further the understanding and treatment of CLP. Future studies should consider including a broader range of databases to provide a more comprehensive global view of CLP research trends.

## Introduction and background

Saudi Arabia, situated in Western Asia, is recognized as the second-largest country in the Arabian region. With a population of approximately 34.81 million, as reported by the General Authority for Statistics in 2020, it ranks as the 41st most populous country globally. The abundance of oil and natural gas reserves in Saudi Arabia has been a significant blessing, propelling remarkable economic development and transforming the nation into one of the world's leading petroleum exporters [1].

The advent of the internet revolutionized the way bibliographical tools are utilized [2]. With the internet becoming accessible to a broader population, researchers, scientists, and scholars gained unprecedented access to a vast amount of information and data [3]. Overall, the accessibility of the internet in the 21st century has transformed the landscape of bibliographical tools and scientometrics [3]. The digital revolution has made these tools more advanced, easily accessible, and user-friendly, empowering researchers with the ability to navigate the vast realm of scholarly information and contribute to the advancement of knowledge [4].

Cleft lip and palate (CLP) is a prevalent craniofacial birth defect, with a significant impact on individuals' physical and functional well-being. It is considered the most common craniomaxillofacial birth defect in humans [5]. CLP is a complex condition that arises because of a combination of genetic and environmental factors, leading to the incomplete development of the lip and/or palate during fetal development [5].

In addition to the functional difficulties in feeding, CLP can also have a significant impact on facial appearance [6]. The visible cleft can cause aesthetic concerns and may affect individuals' self-esteem and social interactions [5]. Surgical interventions are commonly employed to address the physical aspects of CLP, with the goal of achieving a more normal facial appearance and functional outcomes [6]. These procedures typically involve the repair of the lip and palate, often performed in a staged approach over the course of several years [6].

In 2017, a comprehensive analysis of research trends in the field of CLP was conducted over an 18-year period. It applied bibliometric principles to analyze the development and characteristics of CLP-related research. The study retrieved a total of 9,040 articles published between 2000 and 2017 [7]. Additionally, specific information or analysis related to the country of Saudi Arabia or its contribution to the research on CLP [7].

Despite the notable advancement of research and treatment in the field of CLP in Saudi Arabia over the past few decades, the peer-reviewed literature lacks a detailed description of the quality and quantity of Saudi research specifically focused on CLP from a bibliometric perspective. Furthermore, it is important to understand the breadth and depth of CLP research in Saudi Arabia as the healthcare system continues to evolve rapidly in response to demographic shifts and socioeconomic changes, identifying how CLP has been approached in the scholarly literature will provide a foundation for future research, policy-making, and clinical practices. This analysis will also reveal the extent of international collaboration, the most influential studies, and emerging trends in CLP research within the Saudi context. Therefore, the current study aims to explore the trend of publications related to CLP in the Saudi Arabian context. This analysis will shed light on the research output, advancements, and collaboration in the field of CLP in Saudi Arabia, providing valuable insights for further development and improvement of care for individuals affected by this condition.

## Review

Materials and methods

Study Setting and Design

The Web of Science database was selected for this bibliometric analysis because of its comprehensive coverage and robust indexing of scientific literature. We utilized the advanced search engine of the Web of Science as our primary tool for conducting the bibliometric search. This particular search engine was selected because of its capability of providing a standardized dataset for analyzing and monitoring various bibliographical parameters. These parameters include author names, keywords, affiliations, countries, journal titles, number of citations, and broad subject categories. One notable advantage of using the Web of Science is its vast collection of 1.6 billion cited references spanning from 1900 to the present day, making it a comprehensive and reliable resource for our bibliometric analysis [8]. As the data were downloaded from available published research, no ethical approval was required.

Data Extraction

The data extraction was conducted independently by two researchers, Hatem Hamadallah and Khalid Alturki (HH and KA). A standardized data extraction form was developed and piloted on a sample of 10 studies before formal extraction to ensure consistency and resolve any ambiguities in the extraction process. The form was designed to capture relevant information such as study identifiers (author names, year of publication, title, etc.) and main outcomes. Data from studies agreed upon for inclusion was then independently extracted onto the standardized extraction form by HH and KA. A periodic reliability check was conducted by having both researchers extract the same five random studies to ensure accuracy and consistency in data extraction. Any differences were discussed to reach an agreement. If a consensus still could not be reached between HH and KA regarding study selection or data extraction after discussion, the third and fourth researchers (MA and AO) were consulted to make the final determination. Comprehensive records of the screening and extraction process were maintained, including reasons for exclusions at each stage.

Data Analysis

The data extracted from the Web of Science were imported into Microsoft Excel (Microsoft® Corp., Redmond, WA) for initial sorting, analysis, and excluding any irrelevant publication. As a result, we obtained a total of 89 scientific articles relevant to CLP. Bibliometric indicators, such as the H-index, total citation count, and publication count, were utilized to analyze the data. Additionally, the data was visually represented using Biblioshiny. This visualization was facilitated through the utilization of R (R Foundation for Statistical Computing, Vienna, Austria).

Results

Most Cited Documents

The articles that were identified were analyzed to identify the top 10 most cited articles. The results of this analysis are presented in (Table 1). According to the citation criteria used, the article by Sabbagh [9] has received the highest number of citations compared to the other articles. This particular article, titled "Passive Smoking in the Etiology of Non-Syndromic Orofacial Clefts," was published in the *PLoS One*journal (Table 1).

**Table 1:** Top 10 Articles on Cleft Lip and Palate (CLP) in Saudi Arabia Based on the Number of Citations Received

Authors	Citations	Year	Title	Journal
Sabbagh [9]	75	2015	Passive smoking in the etiology of non-syndromic orofacial clefts	PLoS One
Seifeldin [10]	56	2016	Is alveolar cleft reconstruction still controversial? (Review of literature)	Saudi Dental Journal
Alansari [5]	37	2014	Living with cleft lip and palate: the treatment journey	Cleft Palate-Craniofacial Journal
Aljohar [11]	36	2008	Pattern of cleft lip and palate in hospital-based population in Saudi Arabia: retrospective study	Cleft Palate-Craniofacial Journal
Alkofide [12]	34	2008	Sella turcica morphology and dimensions in cleft subjects	Cleft Palate-Craniofacial Journal
Alam [13]	30	2021	Sagittal jaw relationship of different types of cleft and non-cleft individuals	Frontiers in Pediatrics
Nwoku [14]	29	2005	Retrospective analysis of secondary alveolar cleft grafts using iliac or chin bone	Journal of Craniofacial Surgery
Kumar [15]	28	1991	Facial clefts in Saudi Arabia: an epidemiologic analysis in 179 patients	Plastic and Reconstructive Surgery
Sabbagh [16]	26	2015	Birth prevalence of non-syndromic orofacial clefts in Saudi Arabia and the effects of parental consanguinity	Saudi Medical Journal
Al-Namankany [17]	25	2018	Effects of cleft lip and palate on children's psychological health: a systematic review	Journal of Taibah University Medical Sciences

Authors with the Most Articles

**Figure 1:**
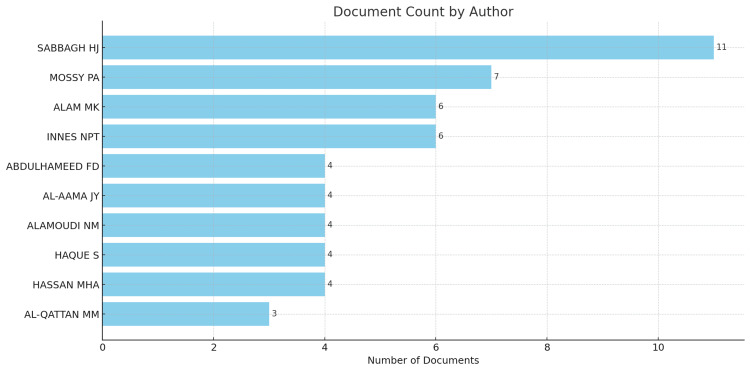
Authors with the Highest Number of Publications on CLP Articles Related to Saudi Arabia

The authors with the most articles were analyzed using Biblioshiny. The results of this analysis are presented in Figure 1. According to the analysis, Sabbagh has the highest number of articles with 14 articles. They are followed by Mossey (seven articles), and Alam and Innes each have six articles (Figure 1).

Institutions with the Most Relevant Articles

**Figure 2:**
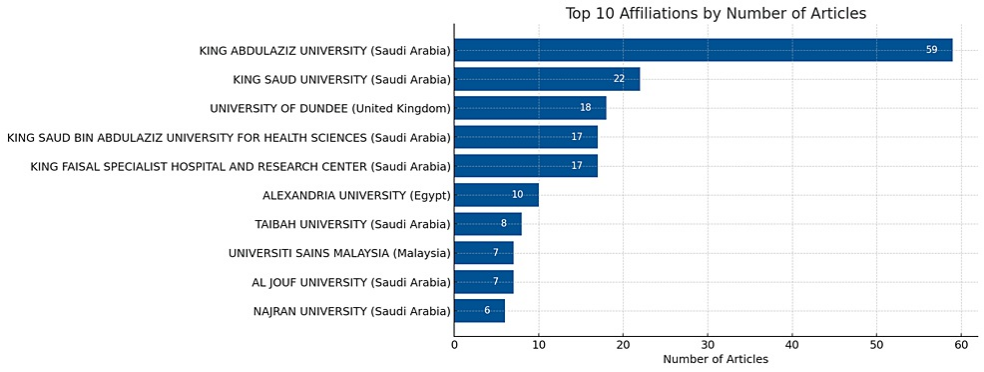
Top 10 Academic and Research Institutions with the Most Saudi Publications on CLP

Based on the bibliometric analysis, the top 10 institutions with the most relevant articles on cleft were listed in (Figure 2). King Abdulaziz University published the highest number of articles on cleft, with a total of 59 articles, followed by King Saud University with 22 articles as shown in Figure 2.

Journals with the Most Relevant Articles

**Figure 3:**
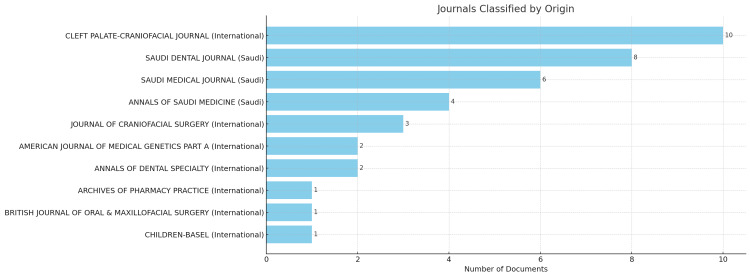
Journals with the Highest Number of Saudi Publications on CLP

After carefully examining the analyzed data, it was determined that the top 10 journals with the highest number of articles related to the topic of cleft, authored by Saudi authors, are presented in (Figure 3). The *Cleft Palate-Craniofacial *journal published 10 articles specifically related to the topic of the cleft. In second place, the *Saudi Dental Journal* had 8 publications focusing on cleft. The *Saudi Medical Journal *secured the third position with six articles published on the same topic (Figure 3).

Yearly Scientific Contribution

**Figure 4:**
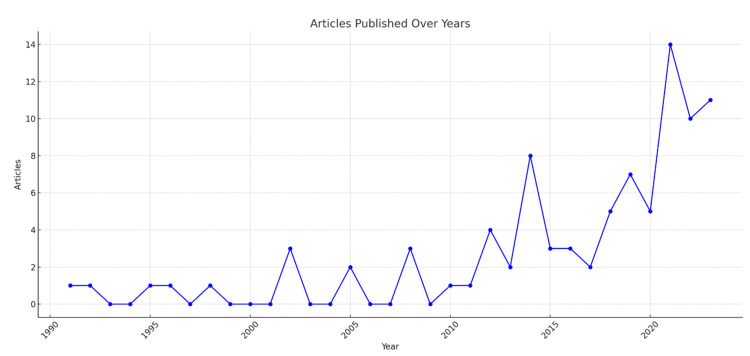
Yearly Scientific Contribution and Trends in Publications on CLP Saudi Research The chart shows the number of scientific articles published in Saudi Arabia on the topic of CLP over the years from 1990 to 2023.

After analyzing the data over a span of time, it is evident that the publication rate experienced significant growth from 2014 to 2023, as demonstrated in (Figure 4). Notably, 2021 emerged as the year with the greatest quantity of articles published across all journals, reaching a peak of 14 publications, as shown in Figure 4.

H-Index

The H-index, also known as the Hirsch index, evaluates the influence of an individual scientist rather than a journal. It is defined as the maximum number of publications by a scientist that received h or more citations each, while the remaining publications have no more than h citations each [18]. While the study primarily focuses on research trends within Saudi Arabia, it benefits from a global perspective through the contributions of international researchers. Not all authors involved in the articles are from Saudi Arabia, indicating a collaborative approach that incorporates insights and expertise from researchers worldwide. Through the analysis of the data, we have identified the top 10 authors with the highest H-index, which are presented in Table 2.

**Table 2:** Top 10 Corresponding Authors in the Field of CLP Research in Saudi Arabia Ranked by H-Index Score The authors listed in this table represent the top individuals who have made influential contributions to cleft lip and palate research based on the H-Index. They are affiliated with institutions in Saudi Arabia, the United Kingdom, and India.

Author Name	H-Index
Mossey, UK	6
Sabbagh, SA	6
Innes, UK	5
Alamoudi, SA	4
Abdulhameed, SA	3
Al-Aama, SA	3
Alam, IN	3
Aljohar, SA	3
Hassan, SA	3
Al Mahdi, SA	2

Citations Per Year

**Figure 5:**
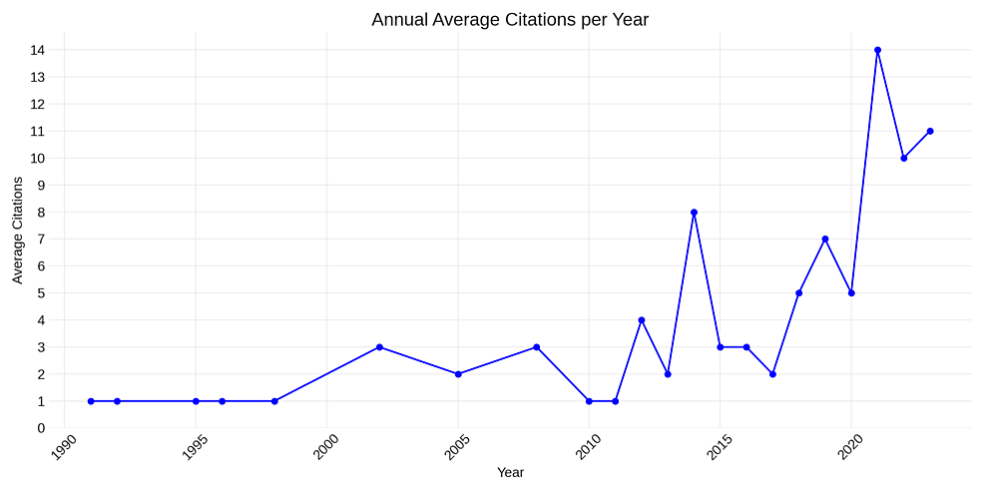
Average Citation Trends Over Time for Publications on CLP Research from Saudi Arabia This figure presents the average number of citations received per year for publications related to CLP that were authored/co-authored by Saudi researchers.

Based on the analysis of the data, it appears that the average number of citations per year experienced significant growth from 2005 to reach its peak in 2015. However, after 2015, the citation count started to decline gradually until 2022, as shown in Figure 5.

Discussion

The current bibliometric analysis aimed to investigate the research trends concerning CLP in Saudi Arabia. The study utilized the Web of Science database as its main data source. While there have been numerous studies conducted globally that discuss the publication trends related to CLP [19-21], this analysis is unique in that it specifically focuses on publications originating from Saudi Arabia. The analysis encompassed a total of 89 articles that were directly relevant to CLP. The main emphasis of this study was to examine the research output and contributions made by Saudi Arabia in the field of CLP.

The results of this bibliometric analysis provide valuable insights into the trends and progress of CLP research originating from Saudi Arabia. The most influential studies were identified based on the number of citations received. Notably, the article by Sabbagh et al. (2015) examining the relationship between passive smoking and non-syndromic CLP has had a significant impact, receiving the highest citation count of 75 citations. This suggests that it has made an important contribution to furthering the understanding of environmental risk factors.

When analyzing author contributions, Sabbagh emerges as the leading researcher in this field based in Saudi Arabia. With the highest number of publications at 14 articles and an H-index of 6, Sabbagh has demonstrated sustained and impactful work in this area over many years. Other top authors include Mossey, Alam, and Innes who each have produced six or more publications, highlighting their roles as prominent researchers driving this field forward.

The findings of this analysis shed light on several important aspects of CLP research in Saudi Arabia. First, the number of publications on CLP in Saudi Arabia has been steadily increasing over the years, reflecting the growing interest and emphasis on this field of study. This upward trend in research output indicates an increasing recognition of the importance of addressing the challenges and improving the care for individuals affected by CLP within the Saudi context.

**Figure 6:**
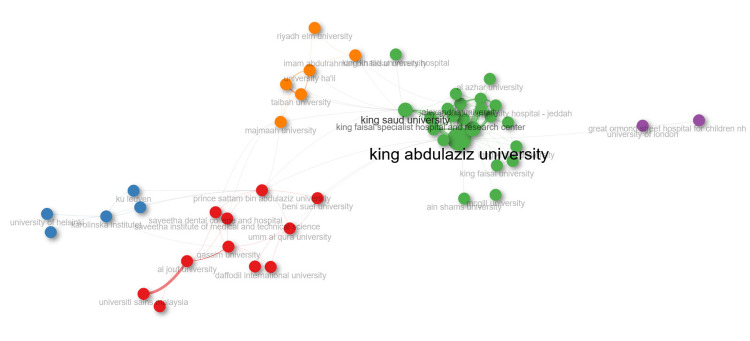
Collaboration Network of Institutions that have Collaborated on CLP Research in Saudi Arabia The figure shows a network of institutions that have collaborated on CLP research in Saudi Arabia. The nodes in the network represent the institutions; the size of the nodes and opacity of the institution name are proportional to the number of publications that the institution has produced on CLP. Image credits: The authors used Biblioshiny to create the figure.

King Abdulaziz University and King Saud University are the two institutions that have collaborated the most on CLP research in Saudi Arabia, as shown in Figure 6. These two institutions are also the ones that have produced the most publications on CLP, as shown in Figure 2. The figure also shows that many other institutions have collaborated on CLP research in Saudi Arabia but to a lesser extent.

**Figure 7:**
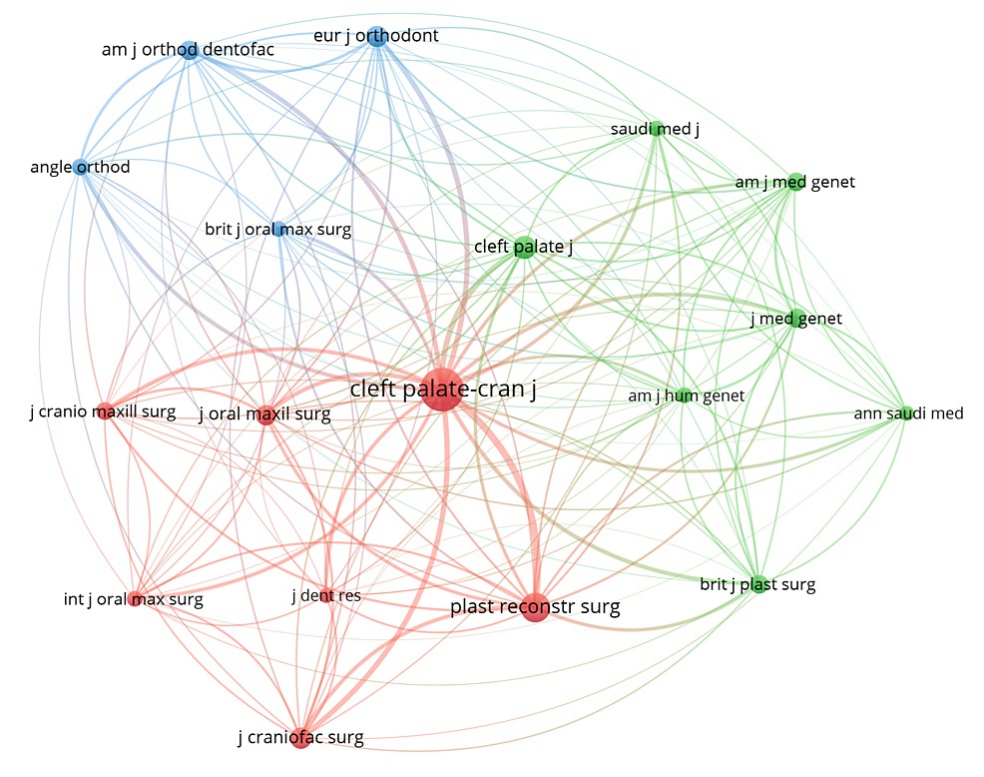
Co-citation Network of the Most Frequently Cited Journals in CLP Saudi Publications The nodes in the map represent the journals, and the edges between the nodes represent the co-citation relationships between the journals. The size of the nodes is proportional to the number of times the journal has been cited, and the thickness of the edges is proportional to the strength of the co-citation relationship between the journals. Image credits: The authors used VOSviewer to create the figure.

The co-citation network presented in Figure 7 provides valuable insights into the journals that are most influential in disseminating research on CLP from Saudi Arabia. The network shows that the *Cleft Palate-Craniofacial Journal and Plastic and Reconstructive Surgery* receive the highest number of co-citations. These two journals form the largest nodes in the network, indicating that they are core sources for Saudi researchers publishing in this field. Other prominently co-cited journals are the *Cleft Palate Journal *and *Journal of Craniofacial Surgery*. This co-citation network highlights the important role played by the *Cleft Palate-Craniofacial Journal*, *Plastic and Reconstructive Surgery*, and the *Cleft Palate Journal *in particular, as leading international journals for disseminating high-quality Saudi research regarding CLP.

**Figure 8:**
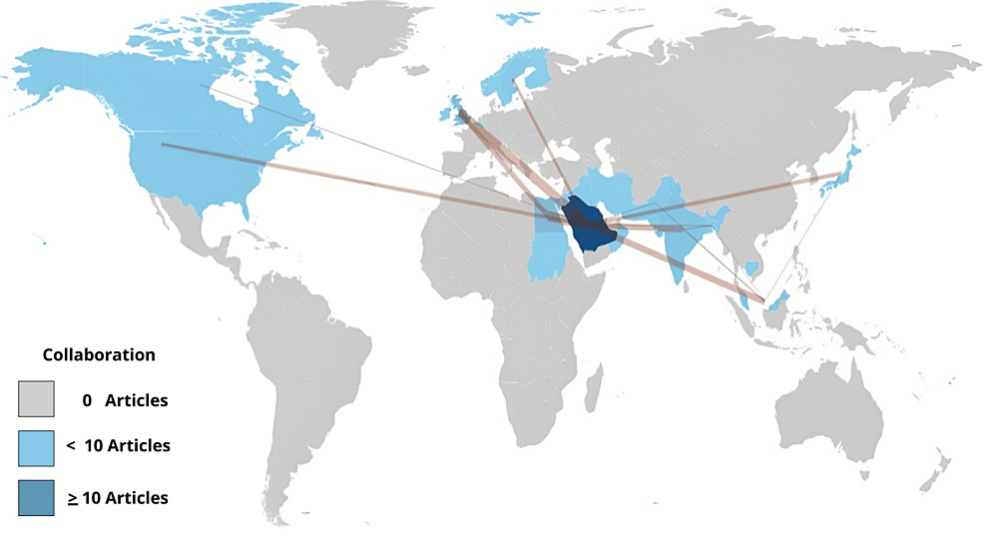
Countries with Collaborations on CLP Research Publications from Saudi Arabia World map visualization with countries color-coded according to countries with collaborations with Saudi Arabia in CLP research. Countries that have coauthored publications with Saudi researchers are colored blue on the map, and the darker shades of blue represent countries that have more extensive collaboration with Saudi Arabia in CLP research. Image credits: The authors used Biblioshiny to create the figure, edited by Khalid N. Alturki.

In addition to the research contributions from Saudi Arabia, collaborative affiliations were observed with countries such as the United States of America, Canada, the United Kingdom, Oman, India, Egypt, Sudan, Japan, Finland, and others, as shown in Figure 8. These collaborations reflect the global nature of CLP research and the exchange of knowledge and expertise among researchers from different countries. The United Kingdom and Egypt have emerged as key partners in research collaborations with Saudi Arabia in the context of CLP research. These collaborations signify the active participation of researchers from the United Kingdom and Egypt in the field of CLP and their commitment to advancing knowledge and finding effective solutions. Such international collaborations can lead to a more comprehensive understanding of CLP and facilitate the development of effective interventions and treatments.

Overall, this bibliometric analysis provides valuable insights into the research trends and contributions in the field of CLP in Saudi Arabia. The increasing research output reflects the growing interest and emphasis on improving the care and understanding of CLP within the Saudi context. The identification of influential articles, authors, institutions, and journals can guide future research endeavors, policymaking, and clinical practices in the field of CLP in Saudi Arabia. Furthermore, the important key factor in conducting this review is the prevalence of CLP in Saudi Arabia, which appears to be increasing [16]. Previous studies have reported the birth prevalence of cleft lip with or without cleft palate in Saudi Arabia to be 2.19/1000 live births, making it one of the highest reported rates locally [22]. A more recent systemic review conducted in 2023 estimated the prevalence to be within the range of 0.65-1.9/1,000 live births [23]. This increasing prevalence over time suggests that CLP is an important public health issue in Saudi Arabia requiring further research attention. Understanding trends in scholarly research output related to CLP from Saudi authors will provide valuable insights into the advancements made within the local healthcare system to address the needs of individuals affected by this condition.

One of the key developments in recent years is the decision by the Ministry of Education to grant access to the Saudi Digital Library (SDL) to all students and professors at Saudi universities [24]. This move has opened up a wealth of educational and research opportunities for academics across the country. By providing access to a wide range of resources, the SDL is expected to facilitate advancements in the education and publishing processes. The availability of SDL and its integration with popular search engines such as PubMed, Web of Science, Scopus, and Google Scholar has made it easier for Saudi academics to conduct bibliometric analysis and study publication volumes in different fields. This improved accessibility is likely to result in an increase in research output across various disciplines. Scholars now have simpler ways to search for relevant literature, which can lead to more comprehensive and in-depth research.

Limitations and recommendations

While this bibliometric analysis acknowledges the limitation of relying on a single Web of Science database, it is essential to recognize the rigorous auditing process that journals undergo on an annual basis within the Web of Science database. This meticulous evaluation ensures that the included journals adhere to high standards of quality and credibility. With an extensive collection of peer-reviewed scientific journals, books, and conference proceedings, Web of Science stands as the largest abstract and citation database available.

Given these factors, the Web of Science database was chosen as the source for data retrieval and analysis in the current study. It provides a comprehensive and reliable dataset that covers a wide range of scientific disciplines. To conduct the analysis efficiently, appropriate software tools were employed to handle the retrieved data effectively and derive meaningful insights.

We recommend similar future analyses to incorporate additional databases/sources to provide an even more robust and comprehensive depiction of research trends.

## Conclusions

This bibliometric analysis provided valuable insights into the research trends on CLP in Saudi Arabia. The findings show that the research output on this topic has steadily increased, reflecting the growing focus on improving the care and understanding of CLP within the Saudi healthcare system. King Abdulaziz University and King Saud University have made significant contributions as the most productive institutions. The *Cleft Palate-Craniofacial Journal *and *Saudi Dental Journal* have published the most articles on this topic from Saudi authors. International collaboration is also evident between researchers in Saudi Arabia and other countries. While this study provides a useful overview based on the Web of Science database, further analysis incorporating additional databases and literature sources could provide an even more comprehensive perspective on research advances on CLP in the Kingdom of Saudi Arabia.
